# Biochemical profile and bioactive potential of thirteen wild folk medicinal plants from Balochistan, Pakistan

**DOI:** 10.1371/journal.pone.0231612

**Published:** 2020-08-18

**Authors:** Alia Ahmed, Amjad Hameed, Shazia Saeed

**Affiliations:** 1 Department of Botany, University of Balochistan, Quetta, Pakistan; 2 Nuclear Institute for Agriculture and Biology (NIAB), Faisalabad, Pakistan; Institute for Biological Research "S. Stanković", University of Belgrade, SERBIA

## Abstract

The recent focus is on the analysis of biological activities of extracts from thirteen folk medicinal plants from arid and semi-arid zones of Balochistan, Pakistan. Only a small proportion of them have been scientifically analyzed. Therefore the present investigation explores the biochemical and bioactive potential of different plant parts. Superoxide dismutase was detected maximum in *Fagonia indica*, (184.7±5.17 units/g), ascorbate peroxidase in *Tribulus pentandrus* (947.5±12.5 units/g), catalase and peroxidase were higher in *Peganum harmala* (555.0±5.0 and 2597.8±0.4 units/g, respectively). Maximum esterase and α-amylase activity was found in *Zygophyllum fabago* (14.3±0.44 and 140±18.8 mg/g, respectively). Flavonoid content was high in *T*. *pentandrus* (666.1±49 μg/ml). The highest total phenolic content and tannin was revealed in *F*. *olivieri* (72125±425 and 37050±1900 μM/g, respectively). The highest value of ascorbic acid was depicted in *F*. *bruguieri* (F.b.N) (448±1.5 μg/g). Total soluble proteins and reducing sugars were detected higher in *P*. *harmala* (372.3±54 and 5.9±0.1 mg/g, respectively). The maximum total antioxidant capacity was depicted in *Tetraena simplex* (16.9±0.01 μM/g). The highest value of lycopene and total carotenoids exhibited in *T*. *terrestris* (7.44±0.2 and 35.5±0.0 mg/g, respectively). Chlorophyll contents were found maximum in *T*. *pentandrus* var. *pterophorus* (549.1±9.9, 154.3±10, and 703.4±20.2 ug/g, respectively). All taxa exhibited anti-inflammatory activity and anti-diabetic potential. *Z*. *eurypterum* seeds exhibited the highest anti-inflammatory potential (96%), along with other taxa indicated (96–76%) activity when compared with the standard drug diclofenac sodium (79%). Seeds of *T*. *pentandrus* (85%) exhibited the highest anti-diabetic activity. The other taxa also exhibited inhibitory activity of α-amylase ranging from (85–69%) compared with Metformin (67%) standard drug. Phytochemical screening revealed that selected taxa proved to be the potential source of natural antioxidants and could further be explored for *in-vivo* studies and utilized in pharmaceutical industries as potent therapeutic agents validating their ethno-pharmacological uses.

## Introduction

Wild medicinal plants are being utilized as folk medicines globally since long before recorded history. Most of the world’s population depends mainly on these herbal medicines to cure various ailments and to reduce risks of chronic human illness, such as inflammation [1, 2]. Currently most prevailing and powerful drugs used were derived from medicinal plants [3, 4], based on their antioxidants originates from their ethnopharmacological utilization [5]. Many medicinal plants extracts constituted various types of chemicals like alkaloids, flavonoids, terpenoids, glycosides, tannins, etc. Each has a property to control a variety of biological and pharmacological activities such as antimicrobial, anti-parasitic, anti-diabetic, antioxidant, anti-inflammatory, and anticholinesterase [6, 7]. Diabetes mellitus is commonly related to metabolic disorders linked with numerous macro and microvascular problems that accelerate morbidity and mortality [8–11]. It has also been revealed that oxidative stress, rise in free radicals, and deterioration of antioxidant defense may mediate the prevalence of diabetes-associated complications in diabetic patients [12, 13]. Many degenerative disorders like rheumatoid arthritis, joints and shoulder inflammation, heart disease, muscular inflammation, asthma, cancer, and inflammation of the gastrointestinal tract are commonly related to inflammatory processes [14, 15].

It has been proved that herbal extracts containing phyto-antioxidants particularly polyphenols, flavonoids, tannins, and other associated compounds have progressive health effects and decrease disease risk. Recently, much attention has been paid to reveal the effects of bioactive compounds and antioxidant potentials of medicinal plants to analyze their pharmacological activities [16, 17]. Therefore, this study explores and compares nineteen biochemical compounds including five pigments for the first time in thirteen species of Zygophyllaceae. Moreover, *in vitro* antioxidant, anti-diabetic and anti- inflammation activities were tested.

The Zygophyllaceae consists of diverse habits of wild flora including succulents, herbs, undershrub, shrubs, and small trees. The habitat of these plants is predominantly desert or saline areas of temperate and tropical regions around the globe [18, 19]. Many taxa of the family are used ethnomedicinally [20, 21].

*Fagonia* L., is a genus of wild, flowering plants of the family, Zygophyllaceae, having about 45 species all over the world. The distribution of the genus includes parts of Africa, the Mediterranean Basin, Asia and parts of the America. In Pakistan, 10 species are reported earlier. Seven species are found during the present study in southern zones of Balochistan Province. *Fagonia* species were collected to determine the presence of phytochemical compounds. Earlier reported phytochemicals were alkaloids, coumarins, flavonols, saponins, triterpenoid saponins, tannins, cardiac glycosides, and rosmarinic acid [22, 23].

*Peganum harmala* L., earlier in Zygophyllaceae now placed in Nitraceae is a perennial herbaceous plant usually 25–60 cm tall having yellowish-white flowers blooming in April to May and commonly dispersed in many regions of the Province. Earlier commonly known phytochemicals from *P*. *harmala* are alkaloids (harmine, harmaline), flavonoids, and anthraquinones [24, 25].

Genus *Tribulus* L. is considered as the most complex genus in Zygophyllaceae because of the enormous number of invalid specific epithets and also of the variations present in various populations. In Pakistan, four species are reported. *Tribulus* species contain a number of steroidal saponins which may account for their use in muscle building, conditioning, and treatment of certain ailments [26]. *T*. *terristrus* is earlier reported for rich phytochemicals steroidal saponins, flavonoids, alkaloids, and lignan amides [27]. Two new furostanol glycosides, named tribufurosides I (**1**) J (**2**), were isolated from the fruits of *T*. *terrestris* [28]. So far, only few species of *Tribulus* have been chemically analyzed [29].

*Zygophyllum* Linn., with about 90 species, grows mainly between Northern Africa and Central Asia particularly in arid and semiarid areas. *Zygophyllum* species have been phytochemically studied leading to the identification of various classes of compounds including triterpenes, flavonoids, saponins, sterols, simple phenolic compounds, and esters [30]. The main chemical constituents described from *Zygophyllum* species are zygophyllin, quinovic acid, and glycosides, which have been shown to have anti-inflammatory and antipyretic activity [31]. *Z*. *simplex* L. is an annual, succulent halophyte plant, it was found that it belongs to genus *Tetraena*, a sister to genus *Zygophyllum* and recently reassigned to such genus and could be referred as *Tetraena simplex* (L.) Beier & Thulin (syn. *Z*. *simplex* L.) (Zygophyllaceae) [32, 33] *T*. *simplex* (syn. *Z*. *simplex* L.) was documented as a traditional remedy for the treatment of dry scaly patches, cleaning the skin, and as an analgesic and anti-inflammatory agent. Phytochemically, it was reported to contain flavonoids, steroids and saponins [32, 34–36].

## Materials and methods

### Plant collection

The selection of medicinal plants was based on ethnobotanical appraisal. Wild plants were collected from different areas of Balochistan, Pakistan. No specific permissions were required for these locations/activities as plants are growing naturally. No protected area and species were disturbed during the fieldwork. Voucher specimens were prepared, identified following the method as used in the flora of Pakistan [37] verified from Pakistan Plant Database (PPD) and submitted in Botanical garden Herbarium of the University of Balochistan, Quetta. Furthermore records were also available in the open herbarium for future reference (www.openherbarium.org). Fresh aerial parts of nine plants, fruits of two species of *Tribulus*, dry aerial parts of thirteen plants, and ten seeds were collected from different ecological zones of Balochistan (Table 1) for biochemical profiling. Fresh plant samples were transported and kept at -20 ºC. Samples were collected, shade dried and stored at room temperature. Experiments were conducted at Plant Breeding and Genetics Division (MAB Lab), Nuclear Institute for Agriculture and Biology (NIAB), Faisalabad, Pakistan.

**Table 1:** List of the selected taxa with geographical coordinates of the collection sites and voucher specimen’s number.

S. No	Plant Name	Plant code	Part used	Voucher No.	Site of collection	Elevation (meter above sea level)
1	*Z*. *propinquum*	*Z*.*p*	DS, DAP	QUETTA000208	Mushkaf	100–350
2	*Z*. *fabago*	*Z*.*f*	DAP, FAP	QUETTA000090	Quetta	1650–1700
3	*Z*. *eurypterum*	*Z*.*e*	DS	QUETTA000157	Nushki	990–1000
4	*T*. *simplex*	*T*.*s*	DAP, FAP	QUETTA000218	Panjgur	950–990
5	*F*. *indica*	*F*.*i*	DAP, FAP	QUETTA000207	Sibi	100–200
6	*F*. *bruguieri*	*F*.*b*.N	DS, DAP, FAP	QUETTA000222	Nushki (Stone)	990–1000
7	*F*. *olivieri*	*F*.*o*	DS, DAP, FAP	QUETTA000221	Nushki (Janglat area)	1000
8	*F*. *bruguieri*	*F*.*b*.P	DS, DAP	QUETTA000220	Panjgur	970
9	*F*. *paulayana *	*F*.*p*	DS, DAP	QUETTA000219	Panjgur	980
10	*F*. *bruguieri*	*F*.*b*.H	DS, DAP	QUETTA000224	Hingol National Park	0–150
11	*T*. *macropterus *	*T*.*m*	DS, DAP	QUETTA000216	Panjgur	970
12	*T*. *pentandrus* var. *pterophorus*	*T*.*p*.*p*	DS, DAP, FS, FAP	QUETTA000215	Nushki	1000
13	*T*. *terrestris*	*T*.*t*	DS, DAP, FS, FAP	QUETTA000085	Quetta	1600–1700
14	*T*. *pentandrus*	*T*.*p*	DS, DAP, FAP	QUETTA000209	Sibi	130–150
15	*P*. *harmala*	*P*.*h*	DS, DAP, FAP	QUETTA000002	Quetta	1600–1700

Dry Seeds = DS, Dry Aerial Parts = DAP, Fresh Seeds = FS, Fresh Aerial Parts = FAP. *Peganum harmala* (P.h), *Tribulus terrestris* (T.t), *T*. *pentandrus*(T.p), *T*. *macropterus* (T. m), *T*. *pentandrus* var. *pterophorus* (T.p.p), *T*. *pentandrus* (T.p), *Zygophyllum fabago* (Z.f), *Z*. *propinquum* (Z.p), *Z*. *eurypterum* (Z.e), *Tetraena simplex* (T.s), *Fagonia indica* (F.i), *F*. *paulayana* (F.P), *F*. *bruguieri* (F.b.P), *F*. *bruguieri*(F.b.N), *F*. *bruguieri* (F.b.H), *F*. *olivieri* (F.o). Vouchers submitted in open herbarium (www.openherbarium.org) list verified by (www.tropicos.org).

#### Chemicals and reagents

Phosphate phosphate, sodium Chloride, acetone, sodium carbonate were purchased from Acros Organics (USA). Methanol (MeOH), Dithiothreitol (DTT), Ethyline Diamine Tetra Acetic acid (EDTA), 2-propanol, Nitro blue tetrazolium chloride (NBT), Guaiacol, α-Naphthyl acetate solution, Fast blue BB, xylenol orange disodium salt, sulfuric acid (H_2_SO_4),_ Sodium chloride (NaCl), ferrous ammonium sulphate, glycerol, *o*-Dianisidine, Potassium acetate, α-amylase enzyme, 3, 5-dinitrosalicylic acid, bovine serum albumin, hydrochloric acid (HCl), hydrogen peroxide H_2_O_2_, sodium acetate, Glacial acetic acid, Aluminium chloride AlCl_2,_ sodium phosphate, and 2, 2'-Azino-Bis-3-Ethylbenzothiazoline-6-Sulfonic Acid (ABTs) were purchased from Sigma-Aldrich (Merck Germany). Polyvinyl Pyrrolidone (PVPP), starch and Acetic acid were purchased from Bio-world GeneLinx International, Inc., USA. Folin-Ciocalteu (FC) Reagent, Bradford dye, Sodium Dodecyl Sulfate (SDS) were purchased from Thermo Fisher Scientific UK. 2, 6-dichloroindophenol (DCIP) was purchased from Millipore Sigma US.

### Extraction of antioxidant enzymes

Phosphate buffer (50 mM, pH 7.8) was used for plants extraction. 0.15 g of each sample was subjected in 1 mL phosphate buffer to ground. Further the mixture was centrifuged at 14,000×g (20 min, 4ºC). Now the supernatant of this extracted plant material used to perform further phytochemical activities. All the data were taken in replicates of three.

### Superoxide dismutase (SOD) assay

The method of [38] was used to determine SOD activity by homogenizing the fresh aerial parts and fruits of selected taxa in phosphate buffer (50 mM, pH 7.8), EDTA (0.1 mM) and DTT (1 mM) following the procedure of [38] and was further analyzed by assessing its property to stop the photochemical reduction of nitro-blue tetrazolium as explicated by [39]. One unit of SOD activity was demarcated as the amount of enzyme causing 50% inhibition of photochemical reduction of nitro-blue tetrazolium. Absorption was measured in Double Beam Spectrophotometer (Hitachi u-2800).

### Peroxidase (POD) assay

The assessment of POD activity was carried out using method [40] with minor changes. The homogenized mixture of the aerial parts and fruits prepared in 1 mL phosphate buffer (50 mM, pH 7.8), EDTA (0.1 mM) and DTT (1 mM). The assay solution contained 535 μl distilled water, phosphate buffer 50 mM (pH 7.0), guaiacol (20 mM), H_2_O_2_ (40 mM) and 15 μl enzyme extract. The addition of enzyme extract initiated the reaction. At 470 nm, absorbance raise was noted at interval of 20 sec. Absorbance change of 0.01 min^−1^ was demarcated as one unit POD activity. Enzyme activity was expressed on the basis of fresh sample weight.

### Catalase (CAT) assay

Catalase activity was measured by homogenizing the aerial parts and fruit samples prepared in phosphate buffer (50 mM, pH 7.8), EDTA (0.1 mM) and DTT (1 mM). CAT was assessed according to the method used by [40]. The activity was measured in a solution containing 59 mM H_2_O_2_ and 0.1 ml enzyme extract. At 240 nm decrease in absorbance was recorded after interval of 20 seconds. Change in absorbance of 0.01 per min defined CAT activity of one unit. Enzyme activity was expressed on a fresh weight basis.

### Ascorbate peroxidase (APX) assay

The assessment of APX activity was carried out following the method used by [38]. Samples were extracted in phosphate buffer (50 mM, pH 7.0). The measurement of APX the assay buffer contained potassium phosphate buffer (200 mM, pH 7.0), EDTA (0.5 M), ascorbate (10 mM), 1 ml of H_2_O_2_ and 50 μl supernatant. At 290 nm, absorbance decrease was noted after every thirty seconds to estimate the oxidation rate of ascorbic acid [41].

### Hydrolytic enzymes

#### Esterase activity

The α-esterase was determined by using the method as suggested by [42]. α-naphthyl acetate was used as substrates. The reaction mixture contained 30 mM α-naphthyl acetate (30 mM), acetone (1%), and phosphate buffer (0.04 M, pH = 7), and enzyme extract. The mixture obtained was incubated for 15 min at 27ºC in dark. After 15 min, 1 mL of staining solution was added (Fast blue BB 1% and SDS 5% with ratio of 2:5) and again incubated in the dark for 20 min at 27 ºC. Absorbance at 590 nm was measured for α-naphthol produced using standard curve, enzyme activity was α-naphthol produced in μM min^−1^ /g wt.

#### Alpha-amylase activity

A modified method for alpha-amylase activity was followed for all plant samples as described by [43].

### Other biochemical parameters

#### Total Oxidant Status (TOS)

The method of [44] was used to determined TOS. The assay is established on ferrous ion oxidation into ferric ion. The presence of oxidants in the sample in acidic medium and ferric ion measurement produced by xylenol orange was measured/observed [45]. The assay is based on two mixtures R1 (stock xylenol orange solution (0.38g in 500μL of 25Mm H_2_SO_4_) 0.4g NaCl, 500 μL glycerol and volume up to 50Ml with 25mM H_2_SO_4,_ sample extract and R2 (0.0317 g o-dianisidine, 0.0196 g ferrous ammonium sulphate (II). Absorption measured at 560nm after 5 minutes by using a spectrophotometer.

#### Pigment analysis

The concentration of lycopene, chlorophyll (*a* and *b*), total chlorophyll, and carotenoids were examined by the method of [46]. Samples (0.2 g) were ground in acetone (80%) and centrifuged at 10,000 g for 5 min. Absorbance measured at 645, 663, and 480 nm by using a spectrophotometer. Partially

#### Total Phenolic Contents (TPC) and tannin

A micro colorimetric assay was used to measure TPC, by using Folin-Ciocalteu (F-C) reagent [47] with some modifications.0.05 g sample was kept in 95% methanol in dark for 48 hours. After 48 hours, the supernatant was taken. 150 μl FC reagent (10%) and 1.2 ml sodium carbonate (700 mM) were added to it. Place this mixture at room temperature for one hour and took reading at 765 nm. Linear regression equation was calculated by using a standard curve of gallic acid at different concentrations. To measure tannin (0.1g) PVPP was added in the above prepared sample, vortexed vigorously and centrifuged again at 14000 g. the absorbance was measured at 765 nm.

#### Determination of total flavonoid content

The assay was determined by a colorimetric method using Quercetin as standard. Take 200 μl sample prepared in 95% methanol extract and phosphate buffer (40 mM, pH 6.8). Added 50 μl AlCl_2_ (10%) 50 μl Potassium Acetate (1M) Incubate the mixture at room temperature for 40 minutes and take the reading at 415 nm absorbance.

#### Total antioxidant capacity

A modified method of TAC was followed as described by [48]. Due to the presence of antioxidants in the sample, ABTS assay represents a decrease of 2, 2-azino-bis (3-ethylbenzothiazoline-6- sulfonate) radical cation ABTS•+ (blue-green in color) into original ABTS (colorless compound). The antioxidants of the sample extract according to their content decolorize the ABTS•+ radical cation. The reaction mixture contained reagent R1 (mixture of sodium acetate buffer solution and glacial acetic acid, pH5.8), sample extract and reagent R2 (mixture of sodium phosphate buffer solution, glacial acetic acid, hydrogen peroxide and ABTs). After 5 min, at wavelength of 660nm, the absorption of each reaction mixture was measured. This analysis used AsA (ascorbic acid) to develop a calibration curve. The results for antioxidant contents found in plant extracts were measured as μM AsA equivalent to one gram.

#### Reducing sugars (sugar content)

Assessment of reducing sugars level in the plant samples was determined by dinitrosalicylic acid method proposed by [49].

### Total soluble protein content

Protein estimation of plant samples was based on quantitative protein analysis described by [50]. Aerial parts and fruits samples were homogenized in potassium phosphate (50 mM, pH 7.0). Supernatant 5μl and NaCl (0.1N) mixed with1.0 ml of Bradford dye. Incubate the mixture for 30 minutes to get a protein-dye complex. Measure the quantity at 595 nm absorbance by a spectrophotometer.

#### Ascorbic acid (AsA)

2, 6-dichloroindophenol (DCIP) method (Hameed et al. 2005) was followed for measurement of ascorbic acid which measures reduced Vitamin C only. In short, each molecule of ascorbic acid converts a DCIP molecule into a reduced 2, 6-dichloroindophenol (DCIPH2) and this conversion can be recorded as reduced absorption at 520 nm. The calibration curve was drawn with the help of known series of ascorbic acid concentrations. Ascorbate concentration in unknown sample was found by calculating simple linear regression equation.

### *In vitro* anti-diabetic activity (enzyme α-amylase inhibition method)

The *in vitro* anti-diabetic activity was determined by assaying the inhibitory activity of the enzyme α-amylase which involves the breakdown of starch to produce glucose [51]. In this method, 1 mL of methanolic extracts of all species were tested separately and thus added to 1 mL of the enzyme α-amylase in a test-tube and incubated for 10 min at 37°C. Then 1 ml of 1% starch solution was added into it and again incubated for 15 min at 37°C. Then 2 ml 3, 5-dinitrosalicylic acid reagent was added into it, in order to terminate the reaction. The reaction mixture was then incubated in a boiling water bath for 5 min and then allowed it to cool at room temperature. The absorbance of the reaction mixture was then measured at 546 nm in a spectrophotometer. The standard (control) of the reaction without the extract represents the 100% enzyme activity. The % age inhibition of enzyme activity of α-amylase was determined by:

$$\%age\;inhibition\;of\;\alpha ‐amylase=\frac{Enzyme\;activity\;of\;control‐Enzyme\;activity\;of\;extract}{Enzyme\;activity\;of\;control}x\;100=1$$

Results compared with standard drug Glucophage (Metformin) Martin Dow Pharmaceutical (Pak) Ltd.

### *In vitro* anti-inflammory activity (protein denaturation method)

The protein denaturation assay was determined using a modified method as described by [52]. Briefly, the reaction mixture (0.5 mL; pH 6.3) consisted of 0.45 mL of bovine serum albumin (5% aqueous solution) and 0.05 mL of distilled water. The pH was adjusted to 6.3 using a small amount of 1 N HCl. 1 mL of acetone or aqueous extract with final concentrations of (0.1 to 0.5 mg/mL) was added to the reaction mixture. These were incubated at 37°C for 30 min and then heated at 57°C for 5 min. After cooling the samples, 2.5 mL of phosphate buffer solution (pH 6.4) was added. The turbidity was measured by a spectrophotometer at 660 nm. For the negative control, 0.05 mL of distilled water and 0.45 mL of bovine serum albumin were used. Diclofenac sodium with the final concentration of 100, 200, 300, 400, and 500 *μ*g/mL was used as reference drug. The percentage inhibition of protein denaturation was calculated by using the following formula:

$$\%\;age\;inhibition=\frac{\left[Abs\;control‐Abs\;sample\right]}{Abs\;control}x\;100=2$$

Results compared with standard drug Diclofenac sodium (Diclofenac (Na) Getz Pharma Pakistan (Pvt) Ltd.

### Statistical analysis

Data was recorded in mean ± SEM. Resulting data were analyzed by applying descriptive statistics. Two-way ANOVA with three replications was used in analyses. The significance of data was tested by analysis of variance and Tukey (HSD) test at p<0.001using XL-STAT software version 2012.1.02, Copyright Addinsoft 1995–2012 (http://www.xlstat.com).

## Results and discussion

The present study is based on the analysis of biological activities of extracts from thirteen plant species of Zygophyllaceae, remarkably important angiosperm family with many taxa being used in folk medicines. There is a scarcity of data in the literature about these plants. As such making a comparison of the results obtained in the present studies was difficult. Nonetheless, a few papers reported some biological activities of *T*. *terrestris*, *P*. *harmala*, and few species of *Fagonia*. The present investigation explores the presence of enzymatic constituents such as SOD, POD, APX, CAT, esterase, alpha amylase, non-enzymatic antioxidants, and other phytochemicals like AsA, TOS, TAC, TSP, TPC, TF, tannins and pigments. Selected plants also provided evidence for the anti-diabetic and anti-inflammatory potential of varying extent in seeds and aerial parts. The difference in pro-oxidants and antioxidants causes oxidative stress and chronic diseases in the body [53]. Cellular damage results in causing cancer. One of the mechanisms behind the anti-oxidation is free radical scavenging action [54]. POD helps in scavenging the reactive oxygen species (ROS), causing cell oxidative injury [55]. The highest values of peroxidase and catalase were depicted in the aerial parts of *P*. *harmala* (2597.8±0.4 units/g f. wt. and 555.0±5.0 units/g f. wt., respectively) as shown in S1a and S1B Fig, respectively. Plant species having high antioxidant activities can be utilized for different therapeutic applications for the treatment of oxidative stress-induced diseases.

The highest APX value was recorded in *T*. *pentandrus* (947.5±12.5 units/g f. wt.) S1D Fig. Ascorbate peroxidase (APX) enzyme is crucial for the protection from damage by H_2_O_2_ and hydroxyl radicals (•OH) [56]. Antioxidant enzymes activities namely, CAT, POD, and SOD were not tested earlier in selected wild medicinal taxa while few records reported earlier in other wild taxa like *Rumex obtusifolius* a wild medicinal plant and found to have a good antioxidant capacity [57]. Similarly, *Calamintha officinalis* also has potent antioxidants [58]. Alpha-amylase and esterase activities were higher in *Z*. *fabago* (140±18.8 mg/g. and 14.3±0.44 μM/min/g f. wt. respectively) S1e and S1F Fig, respectively. Esterase plays an important role in the disintegration of natural constituents and industrial pollutants and other toxic chemicals. It is also beneficial for the production of optically pure compounds, perfumes, and antioxidants [59].

The antioxidant enzyme catalase is present in all animal tissues with its highest activities in liver and red blood cells which defend the tissues from highly reactive hydroxyl radicals by decomposing hydrogen peroxide. Decrease amount of catalase causes numerous damages due to hydrogen peroxide and superoxide radical assimilation [60]. Superoxide dismutase enzyme referred as the significantly involved in cellular defense, therefore it is considered as an indicator of antioxidant capacity [61]. The highest value of superoxide dismutase (184.7±5.17 units/g f. wt.) was observed in *F*. *indica* shown in S1C Fig. Traditionally this plant is used for anticancer treatment and possibly detected high concentrations of SOD and TAC may be responsible for this therapeutic effect.

Maximum TAC was depicted in fresh samples of *T*. *simplex* (16.9±0.01μM/g. f. wt.) followed by *F*. *indica* (15.6±0.04μM/g. f. wt.) shown S2A Fig. No significant variation was detected among all dry aerial parts of selected plants. In general maximum TAC was detected in the seeds of *Z*. *eurypterum* (15.8±2.2 μM/g. dry wt.) followed by the aerial parts of *T*. *simplex* (15.7±2.33 μM/g.dry wt.). In the seeds of the selected taxa, no significant variation was detected the maximum TAC was in *F*. *olivieri* (15.6±2.4 μM/g. s. wt.). The second highest value was in *F*. *bruguieri* (F.b.N) (15.5±2.5 μM/g. s. wt.) Previously, the aerial parts of *F*. *longispina* were reported to be a good source for natural antioxidants [22]. Previously, *F*. *cretica* was also found to have high antioxidant and radical scavenging potential due to the high TPC and TFC [62]. *F*. *olivieri* can serve as a natural source to develop the free radical scavengers beneficial in the prevention of oxidative stress development [63].

Total flavonoid content (TFC) in the methanolic extract showed maximum quantity in fresh samples of *T*. *pentandrus* (666.1±49μg/ml sample) shown S2B Fig. Fruit samples (fresh) showed the highest value of flavonoid and ascorbic acid in *T*. *terrestris* (566.1 ±5.1 μg/ml sample and 456.5±9.5 μg/g f. wt., respectively). In dry aerial parts of *T*. *pentandrus* flavonoid content gives a maximum amount of TFC (495.4±16.4 μg /mL sample) followed by *F*. *bruguieri* (F.b.H) (395.1±37 μg/mL sample). While in the seeds of *P*. *harmala* showed maximum TFC (418.8±16.7μg/mL sample) followed by *F*. *bruguieri* (F.b.P) (391.8±5.12 μg/mL sample). Flavonoids are considered to be effective free radical scavengers in fruits, vegetables, and medicinal plants. Highest ascorbic acid reported in *P*. *harmala* shown S2C Fig. Ascorbic acid is involved in a number of physiological processes, such as POD and SOD [64].The highest flavonoids and ascorbic acid content in fruits of *T*. *terrestris* and aerial parts of *T*. *pentandrus* in the present study validated its traditional medicine use and may be responsible to cure various ailments. *F*. *olivieri* as shown S2C Fig that gives the highest amount of tannins.

Total soluble protein and reducing sugar were high in fresh aerial parts of *P*. *harmala* shown in S3a and S3B Fig, respectively. Earlier [65] isolated antioxidant protein from *P*. *harmala*. Seeds possessed antioxidant activity, and this activity was due to the presence of hydrophobic amino acids. *P*. *harmala* is one of the most frequently used medicinal plants to treat hypertension and cardiac disease worldwide [66]. The maximum total soluble proteins (168.3±6.3 mg/g dry wt.) were depicted in dry aerial parts of *T*. *simplex*. No significant variation was observed in seed samples, the highest value of total soluble proteins (248.6±30 mg/g s. wt.) was found in *F*. *bruguieri* (F.b.N). Total oxidant status (TOS) was lower in *F*. *indica* (1275±475 μM/g. f. wt.) shown in S3D Fig.

Total flavonoid content was calculated in and expressed as μg/mL in methanolic extract using quercetin as standard (Table 2). Significant difference was observed among all selected species of Zygophyllaceae. In the aerial parts *T*. *pentandrus* flavonoid content gives maximum amount of TFC (495.4±16.4 μg /mL sample) followed by *F*. *bruguieri* (F.b.H) (395.1±37 μg/mL sample). While seeds of *P*. *hermala* showed maximum TFC (418.8±16.7μg/mL sample) followed by *F*. *bruguieri* (F.b.P) (391.8±5.12 μg/mL sample) (Table 3). Total phenolic content (TPC) was estimated, in aerial parts of selected taxa. No significant TPC variation was found among (Table 4). In general, the highest TPC was depicted in *T*. *pentandrus* var. *pterophorus* (63025±1725 μM/g. dry wt.) followed by *F*. *bruguieri* (F.b.N) (54600±1350 μM/g. dry wt.). Seeds of selected plant samples showed significant variation. Highest TPC was detected in *Z*. *propinquum* (69225±775μM/g. s. wt.) followed by *F*. *bruguieri* (F.b.N) (66850±3900 μM/g. s. wt.) as shown in Table 3. No significant Tannin variation was detected among aerial parts as well as in seeds of all tested taxa (Table 2). However, the highest amount of tannins was estimated in *T*. *pentandrus* var. *pterophorus*. (40375±4125 μM/g dry wt.) followed by *Z*. *fabago* (39175±4825 μM/g. dry wt.). In seeds, the highest amount of tannins was estimated in *P*. *hermala* (47525±2575 μM/g s. wt.) followed by *Z*. *propinquum* (42625±175 μM/g. s. wt.) shown in Table 3. Ascorbic Acid was observed, in aerial parts of all selected taxa and significant difference found among the studied taxa (Table 4). The highest value of AsA was found in *F*. *olivieri* (744.2±2.7 μg/g dry wt.) followed by *F*. *bruguieri* (F.b.P). AsA content in seeds showed no significant variation. In general the highest AsA content was found in *F*. *bruguieri* (F.b.H) (740.8±2.19 μg/g s. wt.) given in Table 3. Alpha-amylase activity in dry aerial parts of selected plants was assessed (Table 2) No significant variation was found among the taxa of Zygophyllaceae. However, the highest α-amylase activity was found in *T*. *simplex* (164.9 ±3.39 mg/g. dry wt.) followed by *Z*. *fabago* (153 ± 6.6 mg/g. dry wt.). In seeds of selected taxa significant variation was found among various taxa is given in (Table 3). The highest value was observed in *F*. *bruguieri* (F.b.H) (159.5±11.8 mg/g. s. wt.) followed by *F*. *paulayana* (133.9±0.37 mg/g. s. wt.) given in Table 3. A significant variation of the total soluble protein was observed among all tested taxa dry samples (Table 2). The maximum total soluble proteins (168.3±6.3 mg/g dry wt.) were depicted in *T*. *simplex*. No significant variation was observed in seed samples, the highest value of total soluble proteins (248.6±30 mg/g s. wt.) was found in *F*. *bruguieri* (F.b.N) (Table 3). TAC was measured in the aerial parts of selected taxa of Zygophyllaceae (Table 2). No significant variation was observed among all selected plants. In general, the maximum TAC was found in *Z*. *eurypterum* (15.8±2.2 μM/g. dry wt.) followed by *T*. *simplex* (15.7±2.33 μM/g.dry wt.). In seeds of selected taxa no significant variation was detected the maximum TAC was in *F*. *olivieri* (15.6±2.4 μM/g. s. wt.) while the second highest was in *F*. *bruguieri* (F.b.N) (15.5±2.5 μM/g. s. wt.), Table 3. Reducing Sugar was measured in aerial parts of all selected plants (Table 2) there was a significant difference found among all taxa. The highest value of reducing sugar was recorded in *Z*. *fabago* (7.47±0.2 mg/g. s. wt.) followed by *Z*. *propinquum* with minimum difference (7.19 ±0.55 mg/g. s. wt.). In seeds, the highest value of reducing sugar was found in *T*. *terrestris* (7.9±0.1 mg/g. s. wt.) as indicated in Table 3.

**Table 2:** Phytochemical analysis in the dry aerial parts of the selected taxa.

Selected taxa (Dry aerial parts)	Total Flavonoid Content	Total phenolic contents	Tannin	Total soluble protein content	Ascorbic acid content	Reducing Sugars	Amylase	Total Antioxidant Capacity
	Quercetin equlient (μg/mL sample)	uM/g. dry wt.	uM/g. dry wt.	mg/g dry wt.	ug/g dry wt.	mg/g. dry wt.	mg/g. dry wt.	μM/g. dry wt.
*P*. *harmala*	361.33±4.51^bc^	46175±2275^a^	37975±1625^a^	73.67±4.33^bc^	714±4^abc^	6.8±0.16^abc^	76.98±2.64^a^	15.55±2.51^a^
*T*. *simplex*	289.77±3.98^bcde^	49575±5375^a^	35600±550^a^	168.33±6.33^a^	638.5±8^d^	5.42±0.28^cd^	164.91±3.4^a^	15.72±2.34^a^
*Z*. *propinquum*	382.8±14.67^bcd^	54775±4575^a^	32300±150^a^	60±6.67^bc^	706.5±4^abc^	7.19±0.55^ab^	97.92±2.83^a^	14.61±3.45^a^
*Z*. *fabago*	301.96±2.39^b^	45150±2850^a^	39175±4825^a^	168±3.33^a^	729.5±13.5^c^	7.47±0.28^a^	153.02±6.6^a^	15.36±2.69^a^
*T*. *pentandrus* var. *pterophorus*.	203.11±8.84^de^	63025±1725^a^	40375±4125^a^	109±0.33^abc^	697.25±0.75^c^	5.1±0.12^cd^	77.92±4.72^a^	15.22±2.84^a^
*T*. *terrestris*	178.99±24.65^de^	47750±1650^a^	34650±1500^a^	101.33±0.67^bc^	680.75±1.75^c^	5.26±0.2^cd^	90.94±11.32^a^	14.61±3.45^a^
*T*. *pentandrus*	495.43±16.42^a^	50700±1700^a^	38150±2450^a^	91.33±4^bc^	686.5±10.5^c^	6.13±0.2^cd^	98.3±9.62^a^	13.9±4.16^a^
*F*. *bruguieri* (F.b.H)	395.12±37.08^b^	48325±1670^a^	37662.5±742^a^	115.83±9.79^ab^	722.63±2.38^ab^	5.91±0.28^cd^	91.23±18.17^a^	14.94±1.86^a^
*F*. *bruguieri* (F.b.P)	273.08±24.56^cde^	51175±7175^a^	35900±450^a^	53.33±13.33^c^	736.5±3.5^ab^	4.82±0.08^d^	96.98±26.42^a^	15.5±2.56^a^
*F*. *indica*	334.03±33.21^e^	54150±5400^a^	38875±75^a^	110.67±18.67^abc^	733±8.5^ab^	5.34±0.04^cd^	76.42±2.08^a^	15.51±2.54^a^
*F*. *bruguieri* (F.b.N)	199.93±3.18^cde^	54600±1350^a^	37075±525^a^	78.67±2^bc^	733.25±2.25^ab^	5.1±0.04^cd^	88.3±12.45^a^	15.57±2.48^a^
*F*. *paulayana*	143.21±4.24^e^	47750±550^a^	38575±775^a^	108±11.33^abc^	725±5^ab^	5.45±0.16^bcd^	80±1.51^a^	15.6±2.46^a^
*F*. *olivieri*	336.68±18.73^cd^	43975±475^a^	38325±1275^a^	122.33±1.67^ab^	744.25±2.75^a^	5.02±0.2^d^	77.17±1.32^a^	14.36±3.69^a^

Data are presented as mean values ± standard error (n = 3). Statistical analysis: ANOVA test and Tukey (HSD). The different letters above the values in the same column indicate significant differences with Tolerance: 0.0001. Values with the same superscript letters in the same column are not significant.

**Table 3:** Phytochemical analysis in the seeds of the selected taxa.

Selected taxa (Seeds)	Total Flavonoid Content	Total phenolic contents	Tannin	Total soluble protein content	Ascorbic acid content	Reducing Sugars	Amylase	Total Antioxidant Capacity
	Quercetin equlient (μg/mL sample)	uM/g. seed wt.	uM/g. seed wt.	mg/g seed wt.	ug/g seed wt.	mg/g. seed wt.	mg/g. seed wt	μM/g. seed wt
*P*. *harmala*	241.27±139.4^a^	63400±1350^ab^	47525±2575^a^	235.33±80^a^	700.25±45.75^a^	4.19±0.87^c^	130.19±0.38^abc^	15.33±2.73^a^
*Z*. *propinquum*	393.13±247.27^a^	69225±775^a^	42625±175^a^	112.67±0^a^	737.75±0.25^a^	4.03±0.4^ab^	132.83±2.26^bc^	15.27±2.79^a^
*Z*. *eurypterum*	153.02±4.88^a^	44850±135^ab^	36500±1000^a^	48.67±2^a^	686.5±0.5^a^	5.26±0.04^a^	112.64±2.08^bc^	15.84±2.21^a^
*T*. *pentandrus* var. *pterophorus*.	365.84±34.98^a^	51225.00±1475^ab^	39300±550^a^	103.67±16.33^a^	739.50±2.5^a^	6.84±0.2^ab^	72.83±1.51^c^	10.4±7.66^a^
*T*. *terrestris*	227.49±71.56^a^	46050.00±50^bc^	37125±2025^a^	154.33±4.33^a^	724.75±15.25^a^	7.94±0.12^a^	131.13±1.7^ab^	11.71±6.35^a^
*T*. *pentandrus*	431.56±208.84^a^	44025±1325^c^	39275±1075^a^	142.67±2^a^	726±4^a^	7.55±0.2^a^	131.51±2.83^ab^	8.37±9.69^a^
*F*. *bruguieri* (F.b.H)	329.39±10.05^a^	64437.5±2887^a^	39062±1111^a^	181.67±29.42^a^	740.88±2.19^a^	4.84±0.09^c^	159.53±11.84^a^	14.67±2.01^a^
*F*. *bruguieri* (F.b.P)	157.79±1.61^a^	46825±3025^bc^	40625±675^a^	237.00±33^a^	737.75±1.75^a^	4.74±0.08^c^	99.81±0.94^bc^	15.47±2.58^a^
*F*. *bruguieri* (F.b.N)	274.98±8.78^a^	58168±1148^c^	353641±352^a^	178.00±1.84^a^	701.55±1.85^a^	4.38±1.06^ab^	118±2.51^bc^	14.19±1.69^a^
*F*. *paulayana*	390.22±147.35^a^	60750±3500^abc^	38300±650^a^	156.00±30.67^a^	710±18^a^	4.35±0.24^bc^	133.96±0.38^ab^	14.9±3.16^a^
*F*. *olivieri*	215.57±25.18^a^	55400±300^abc^	41700±2750^a^	164.67±4.67^a^	719.50±2.5^a^	4.98±0.08^c^	130.38±0.57^abc^	15.62±2.43^a^

Data are presented as mean values ± standard error (n = 3). Statistical analysis: ANOVA test and Tukey (HSD). The different letters above the values in the same column indicate significant differences with Tolerance: 0.0001. Values with the same superscript letters in the same column are not significant.

The lycopene content in the dry aerial parts of various taxa was measured (Table 4). The highest value was found in *T*. *pentandrus* var. *pterophorus* (9.25± 1.8 mg/g dry wt.) followed by *T*. *terrestris* (8.12±0.01 mg/g dry wt.). Lycopene content in the seeds of various taxa of Zygophyllaceae was investigated (Table 5). The higher value of lycopene was found in *F*. *paulayana* (5.87±0.75 mg/g s. wt.) followed by *F*. *bruguieri* (F.b.N) (4.92±0.19 mg/g s. wt.). The chlorophyll a content was estimated in dry aerial parts of dry samples of various taxa of Zygophyllaceae (Table 4). The chlorophyll a content ranged from maximum in *T*. *pentandrus* var. *pterophorus* (572.1±0.05 ug/g dry wt.) followed by *T*. *terrestris* (548.8±0.2 ug/g dry wt.) to minimum with significant difference in *Z*. *eurypterum* (92.5±8.0 ug/g dry wt.). Chlorophyll a content in the seeds of various taxa (Table 5) ranged from maximum in *F*. *bruguieri* (F.b.N) (197±0.76 ug/g s. wt.) followed by *F*. *paulayana* (171.2±5.3 ug/g s. wt.). The chlorophyll b content was assessed in the dry aerial parts of different taxa of Zygophyllaceae (Table 4). The chlorophyll b content varied among various taxa. The chlorophyll b content ranged from maximum in *T*. *pentandrus* var. *pterophorus* (228 ±63 ug/g dry wt.) followed by *T*. *terrestris* (170.2±1.6 ug/g dry wt.). In the seeds of different taxa, chlorophyll b content varied ranged from maximum in *F*. *paulayana* (70.7±11.2 ug/g s. wt.) next highest was *F*. *bruguieri* (F.b.N) (38±4.6 ug/g s. wt.) to minimum in *Z*. *propinquum* (2.9±0.01ug/g s. wt.) with a significant difference (Table 5). Total carotenoids were investigated in the dry aerial parts of dry samples of different species of Zygophyllaceae (Table 4). Significantly, the highest value of total carotenoids was found in *T*. *pentandrus* var. *pterophorus* (38.6±0.6 mg/g dry wt.) followed by *T*. *terrestris* (38.2±0.02 mg/g dry wt.). In seeds samples significantly the highest value of total carotenoids was found in *F*. *bruguieri* (F.b.N) (19.3±0.00 mg/g s. wt.) (Table 5) followed by *F*. *paulayana* (16.6±1.5 mg/g s. wt.). The total chlorophyll content of the dried aerial parts was measured among different taxa of Zygophyllaceae is shown in (Table 4). Maximum Content of total chlorophyll was depicted in *T*. *pentandrus* var. *pterophorus* (800±62.9 ug/g dry wt.) with significant difference. It was followed by *T*. *terrestris* (719±1.8 ug/g dry wt.). In seeds, the total chlorophyll was measured among different taxa of Zygophyllaceae is given in (Table 5). A significant variation was observed among various taxa. The maximum content of total chlorophyll was depicted in *F*. *paulayana* (242±16.6 ug/g s. wt.). The second highest in *F*. *bruguieri* (F.b.N) (235.3±3.9 ug/g s. wt.). The liquid chlorophyll supplements are important in enhancing energy, detoxification of liver and stomach and colon, eliminating the body and mouth odor, helping in anemia, and aiding in the elimination of mould from the body. The carotenoids are vital because of their strong colours, antioxidant activity as well as their role as precursors of vitamin A. These can be used as safe chemicals for nutraceutical purposes and food supplementation [67].

**Table 4:** Pigment analysis in the dry aerial parts of the selected taxa.

Selected Taxa	Lycopene	Chlorophyll a	Chlorophyll b	Total carotenoids	Total chlorophyll
	mg/g dry wt.	ug/g dry wt.	ug/g dry wt.	mg/g dry wt.	ug/g dry wt.
*P*. *harmala*	6.02±0.03^ab^	401.97±6.6^ab^	76.68±0.5^ab^	30.37±0.34^abc^	478.65±7.11^abcd^
*T*. *simplex*	1.49±0.13^b^	186.81±5.96^bc^	0^b^	9.09±0.64^cde^	183.27±8.63^cd^
*Z*. *propinquum*	1.33±0.03^b^	151.05±0.05^bc^	10.25±1.16^b^	8.64±0.06^de^	161.30±1.11^cd^
*Z*. *fabago*	7.10±0.61^ab^	418.84±31.71^bc^	121.53±17.67^ab^	31.18±1.98^ab^	540.37±49.38^abc^
*T*. *pentandrus* var. *pterophorus*.	9.26±1.39^a^	572.10±0.05^a^	228.00±63.01^a^	38.63±0.62^a^	800.11±62.96^a^
*T*. *terrestris*	8.13±0.01^ab^	548.87±0.24^a^	170.22±1.61^ab^	38.27±0.02^a^	719.09±1.85^ab^
*T*. *pentandrus*	4.55±0.14^ab^	364.44±1.96^ab^	52.87±4.98^ab^	22.52±0.1^abcde^	417.31±3.02^abcd^
*F*. *bruguieri* (F.b.H)	2.76±0.34^ab^	215.16±33.96^bc^	32.41±3.43^b^	15.03±1.94^bcd^	247.57±35.39^cd^
*F*. *bruguieri* (F.b.P)	6.58±0.24^ab^	519.24±5.37^a^	168.96±11.83^ab^	29.94±0.5^abcd^	688.20±17.20^ab^
*F*. *indica*	4.29±0.42^ab^	316.84±23.83^ab^	57.49±8.21^ab^	21.51±1.83^abcde^	374.33±32.03^abcd^
*F*. *bruguieri* (F.b.N)	7.34±3.94^ab^	336.84±109.62^abc^	126.09±81.96^ab^	25.72±10.08^abcde^	462.93±191.58^abcd^
*F*. *paulayana*	4.37±1.02^ab^	308.70±5.17^abc^	61.42±13.13^ab^	18.55±1.96^abcde^	370.11±7.96^abcde^
*F*. *olivieri*	5.98±1.12^ab^	461.19±68.62^a^	121.54±38.51^ab^	27.40±4.76^abcde^	582.72±107.13^abc^

Data are presented as mean values ± standard error (n = 3). Statistical analysis: ANOVA test and Tukey (HSD). The different letters above the values in the same column indicate significant differences with Tolerance: 0.0001. Values with the same superscript letters in the same column are not significant.

**Table 5:** Pigment analysis in the seeds of the selected taxa.

Selected Taxa (seeds)	Lycopene	Chlorophyll a	Chlorophyll b	Total carotenoids	Total chlorophyll
	mg/g seed wt.	ug/g seed wt.	ug/g seed wt.	mg/g seed wt.	ug/g seed wt.
*P*. *harmala*	4.69±1.22^ab^	48.39±17.1^c^	28.45±4.58^abc^	12.99±3.19^abc^	76.84±12.52^b^
*Z*. *propinquum*	1.62±0.16^b^	127.97±0.27^abc^	2.93±0.02^bc^	8.68±0.28^abc^	128.76±2.44^ab^
*Z*. *eurypterum*	1.53±0.03^ab^	92.51±8.09^c^	0^b^	6.21±1.47^e^	75.27±16.90^d^
*T*. *macropterus*	4.06±0.19^ab^	129.78±15.86^abc^	29.07±4.28^abc^	12.93±0.84^abc^	158.85±20.14^ab^
*T*. *terrestris*	3.30±0.15^ab^	106.12±3.82^bc^	17.18±3.17^bc^	10.80±0.2^abc^	123.30±6.99^ab^
*T*. *pentandrus*	2.00±0.66^ab^	114.23±25.31^abc^	7.28±0.5^bc^	7.30±3.2^bc^	103.79±43.53^b^
*F*. *bruguieri* (F.b.H)	1.30±0.36^b^	112.37±9.01^bc^	0^bc^	6.68±0.86^c^	112.84±10.58^b^
*F*. *bruguieri* (F.b.P)	0.96±0.01^b^	149.24±1.55^ab^	0^c^	6.61±0.07^c^	147.71±2.81^ab^
*F*. *bruguieri* (F.b.N)	4.92±0.19^ab^	197.34±0.77^a^	38.04±4.68^ab^	19.38±0^a^	235.38±3.91^a^
*F*. *paulayana*	5.88±0.75^a^	171.25±5.33^ab^	70.80±11.27^a^	16.68±1.57^ab^	242.05±16.61^a^
*F*. *olivieri*	3.58±1.08^ab^	129.12±14.36^abc^	30.48±16.66^abc^	11.34±2.33^abc^	159.60±31.02^cd^

Data are presented as mean values ± standard error (n = 3). Statistical analysis: ANOVA test and Tukey (HSD). The different letters above the values in the same column indicate significant differences with Tolerance: 0.0001. Values with the same superscript letters in the same column are not significant.

The pigment analysis in fresh samples shown in S4A, S4B, S4C, S4D, and S4E Fig shows the significant variation among the selected taxa. The chlorophyll contents (a, b and total), were higher in aerial parts of *T*. *pentandrus* var. *pterophorus* followed by *T*. *terrestris*, whereas carotenoids and lycopene were observed maximum in *T*. *terrestris* followed by *T*. *pentandrus* var. *pterophorus*. The main carotenoids such as zeaxanthin, b-carotene, canthaxanthin, astaxanthin as well as lycopene are prepared synthetically in nutraceutical industry [68]. Furthermore, Lycopene is suggested as one of the efficient carotenoids group for quenching ability. The plants used in the current study detected pigments in Zygophyllaceae taxa can be characterized for the above mentioned applications which can be utilized as supplements or medicines.

This study was carried out to assess the anti-inflammatory and anti-diabetic potential of naturally occurring medicinal plants of Zygophyllaceae from desert and semi-desert areas of Balochistan, Pakistan. The protein denaturation assay was followed for *in vitro* anti-inflammatory activity while anti-diabetic activity was determined by α-amylase inhibitory assay for 13 extracts of aerial parts and 12 seeds extracts. The selected plants also used in traditional medicines by the folks in Balochistan. The selection of the plants was based on ethnobotanical appraisal of local communities, their uses in folk medicines to cure various ailments like fever, cough, inflammation of organs, gonorrhea, urinary tract infection (UTI), diabetes, cancer, etc. the previously secondary metabolites such as flavonoids and alkaloids derivatives were evaluated in Zygophyllaceae [69, 70].

**Fig 1:**
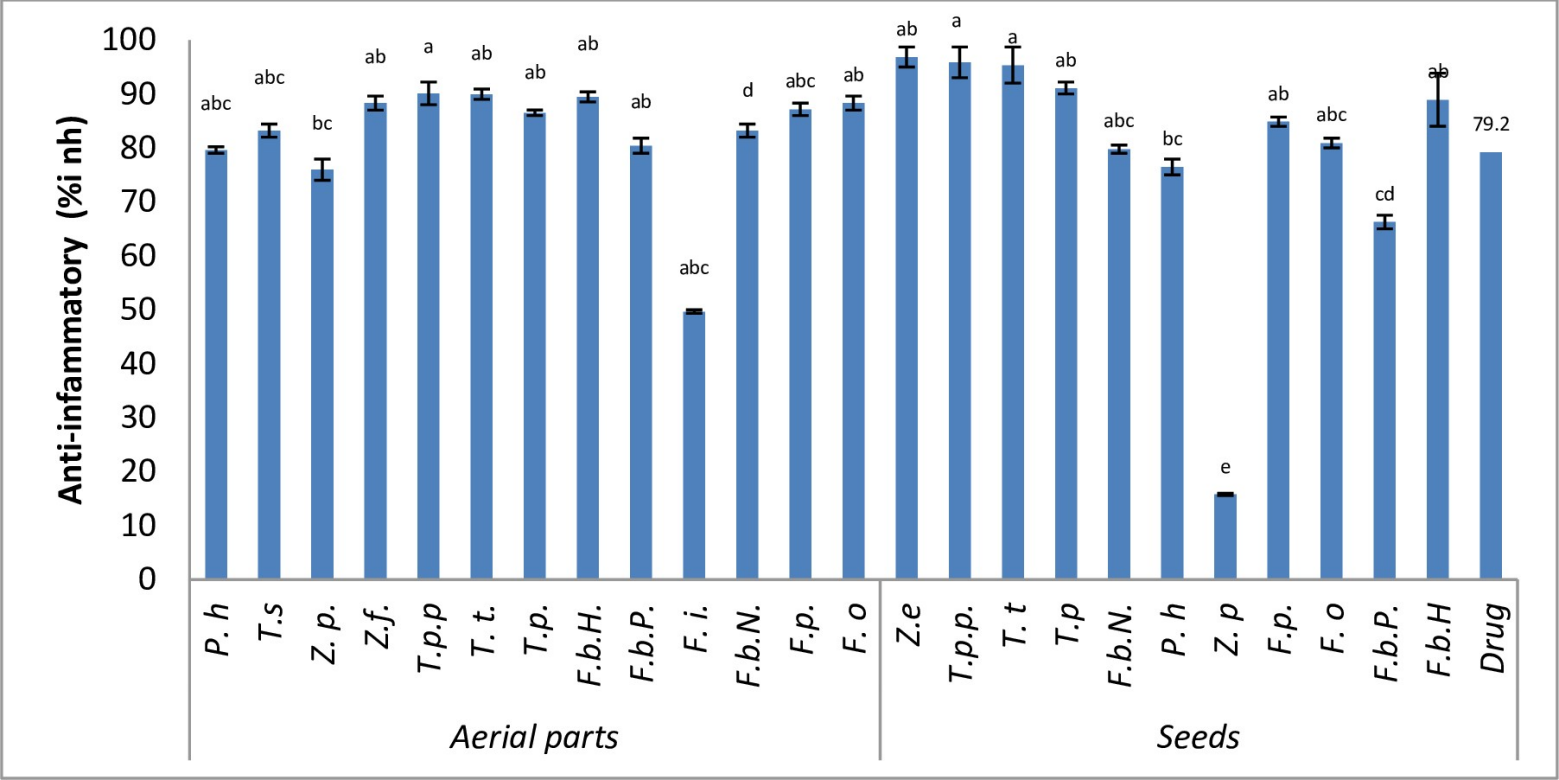
Comparison of anti-inflammatory activity among different taxa. Data are presented as mean values ± SEM (n = 3). Statistical analysis: ANOVA test and Tukey (HSD). The different letters above the values in the same column indicate significant differences with Tolerance: 0.0001.

Inflammation in medical terms is demarcated as a pathophysiological procedure described by soreness, fever, swelling of body parts, loss of function and discomfort [71]. The inhibitory effect among different plants of Zygophyllaceae on albumin denaturation is shown in (Fig 1). Significant inhibition of albumin was observed among different taxa. *Z*. *eurypterum* seeds depicted maximum inhibition with the highest value (96.85±1.85% Inh.). Seeds of *T*. *pentandrus* var. *pterophorus* revealed the next highest value (95.85±2.85%), Seeds of *T*. *terrestris* exhibited the (95.35±3.35% Inh.) activity. *T*. *pentandrus* depicted (91.1±1.1% of Inh.). Earlier in the fruit of *T*. *terrestris* significant anti-inflammatory activity was evaluated [72]. All species of *Fagonia* also exhibited anti-inflammatory activity significant and comparable with the standard drug diclofenac sodium. In the seeds extracts, the highest value was found in *F*. *bruguieri* (F.b.H) (88.9 ± 4.9% Inh). *F*. *paulayana* showed (84.8±0.8%) of inhibition. *F*. *olivieri* and *F*. *bruguieri* (F.b.N) showed (80.9 ±0.9 and 79.7±0.7% Inh., respectively). The seeds of *P*. *harmala* constituted (76.4±1.4% Inh.) activity for inhibition. The seeds of *F*. *bruguieri* (F.b.P) (66.2±1.2% Inh.) revealed less inhibition when compared with the standard drug. The previously anti-inflammatory activity of *F*. *cretica* was studied by [73]. A minimum inhibition of albumin was observed in the seeds of *Z*. *propinquum* (15.79±0.20% Inh.) in comparison with all studied taxa. Previously, *Nitraria schoberi* from Zygophyllaceae fruit extract has been found to have anti-inflammatory effects [74] that are quite in line with the presently observed activities for the seeds and the aerial parts. The aerial parts of all the studied taxa of Zygophyllaceae also exhibited a significant inhibition of albumin. *T*. *pentandrus* var. *pterophorus* revealed the maximum inhibition (90.1±2.1% Inh.) followed by *T*. *terrestris* and *F*. *bruguieri* (F.b.H) (89.9±0.9 and 89.4±0.9% Inh., respectively) anti-inflammatory inhibition. *Z*. *fabago* and *F*. *olivieri* indicated (88.3 ± 1.3% Inh.) inhibition in both plants. *F*. *paulayana* (endemic to the region) revealed (87.1±1.1% Inh.) albumin inhibition activity. *T*. *pentandrus*, *T*. *simplex* and *F*. *bruguieri* (F.b.P) exhibited inhibition activity as (86.5 ± 0.5, 83.2 ±1.2 and 80.4 ±1.2% Inh. respectively). *P*. *harmala* and *Z*. *propinquum* aerial parts also showed a significant inhibition as compared with the standard drug (79.6±0.6 and 75.9±1.9% Inh.). *F*. *indica* (49.6±0.3% Inh.) depicted less inhibition when compared with all other taxa as well as the standard drug. Previously, chloroform and methanol extracts of the aerial parts of *T*. *terrestris* exhibited a significant anti-inflammatory activities at a dose of 200 mg/kg [72]. All examined *Fagonia* spp. in the present study exhibited therapeutic potential especially *F*. *bruguieri* (F.b.H) suggested to be used as an anti-inflammatory as well as an anti-diabetic agents authenticating its folk use. Earlier, *F*. *cretica* was identified for its high therapeutic effects against various types of hematological, liver disorders, neurological and inflammatory conditions. Furthermore, aqueous extract is one of the seventeen ingredients in Norm acid syrup used in the treatment of high acidity and gastritis [75]. Also the endemic taxa of the region i.e. *F*. *paulayana* exhibited high anti-inflammatory potential. Previously, *F*. *longipina* traditionally was used as a preventive for cancer, also used for the treatment of inflammation of the urinary tract [22]. *F*. *schweinfurthii* plant extract gel could be developed as a therapeutic agent for wound healing and anti-inflammatory properties [76].

**Fig 2:**
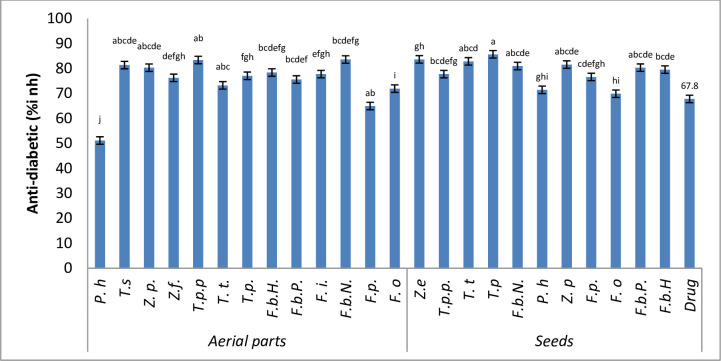
Comparison of the anti-diabetic activity of the different taxa. Data are presented as mean values ± standard deviation (n = 3). Statistical analysis: ANOVA test and Tukey (HSD). The different letters above the values in the same column indicate significant differences with tolerance: 0.0001.

The anti-diabetic activity was analyzed by using amylase inhibition assay. The seeds and the aerial parts of the selected species revealed significant differences in anti-diabetic activity when compared with the standard drug metformin (Fig 2). The seeds of *T*. *pentandrus* (85.65±0.34% Inh.) exhibited the highest anti-diabetic activity among all the selected species. It was followed by the seeds of *Z*. *eurypterum* (83.63±0.63% Inh.), next highest value was found in *T*. *terrestris* (82.8±0.1%). The seeds of *Z*. *propinquum* indicated (81.5±0.4%) activity. *F*. *bruguieri* (F.b.N) depicted (80.9±1.9%) anti-dibetic activity. *F*. *bruguieri* (F.b.P) revealed (80.3±0.6%) enzyme inhibition in the seeds of *F*. *bruguieri* (F.b.H) (79.5±1.0%) inhibition was found. The seeds of *T*. *pentandrus* var. *pterophorus* reported (77.7±0.2%) enzyme inhibition activity. The endemic plant of the region *F*. *paulayana* depicted (76.5±0.4%) anti-diabetic activity. *P*. *harmala* showed (71.4±1.4%) anti-diabetic activity. Seeds of *F*. *olivieri* exhibited (69.8±0.8%) enzymatic inhibitory activity. The seeds of *P*. *harmala* were used as an anti-inflammatory and an anti-diabetic agents [77]. The same therapeutic potential of seeds of *P*. *harmala* was attained in the current study.

The aerial parts of the selected taxa also exhibited a significant enzyme inhibition activity when compared with the standard drug. The highest value was found in *F*. *bruguieri* (F.b.N) (83.59±0.40% Inh.). The second highest value was in *T*. *pentandrus* var. *pterophorus* (83.37±0.62% Inh.). The next highest value was found in *T*. *simplex* (81.3±1.3% Inh.) followed by *Z*. *propinquum* (80.2±1.2% Inh.) succulent plants. *F*. *bruguieri* (F.b.H) depicted (78.3±1.1% Inh.) anti-diabetic activity. *F*. *indica* showed (77.7±0.2% Inh.) activities followed by *T*. *pentandrus* (77 ±1.06% Inh.) inhibition. Aerial parts of *Z*. *fabago* shows (76.*2*±1.*2*% Inh.) inhibitory effect. *F*. *bruguieri* (F.b.P) showed (75.5±0.5% Inh.) enzymatic inhibitory activity. The aerial parts of endemic plant *F*. *paulayana* also showed significant inhibition of amylase enzyme (64.9±0.05% Inh.) Earlier reported *F*. *indica* alone or combined with *Aloe vera* can be used as a natural blood glucose lowering agent [78]. In the present research the other examined *Fagonia* species are potentially active therapeutic agents in comparison with *F*. *indica*. *P*. *harmala* and *Z*. *gaetulum* frequently used to treat hypertension and diabetes mellitus [66].

## Conclusion

The phytochemical screening of all the selected of Zygophyllaceae revealed that these plants have significant potential of enzymatic, non-enzymatic activities, and other phytochemicals like flavonoids, total phenolic compounds, tannins, and pigments. All the taxa proved to have natural antioxidants and could not only be used to treat various ailments but also contribute in the prevention of degenerative diseases and manufacturing of new drugs. Research findings could be utilized for isolation of potential phytopharmacological active compounds from these wild medicinal plants for future research. Species such as *Z*. *eurypterum*, *T*. *pentandrus*, *T*. *terresetris*, *F*. *bruguieri* (F.b.P), and *F*. *paulayana* had greater potential for identification, isolation, and purification of novel therapeutic agents.

## Supporting information

S1 Fig Comparison of a) Peroxidase Activity b) Catalase Activity c) Superoxide Dismutase Activity d) Ascorbate Peroxidase Activity e) Alpha-amylase activity f) Esterase activity.
(DOCX)

S2 Fig Comparison of a) Total Phenolic Contents b) Total Flavonoid c) Ascorbic Acid Content. d) Tannin.
(DOCX)

S3 Fig Comparison of a) Total soluble proteins b) Reducing Sugar c) Total Oxidant Status d) Total Antioxidant Capacity.
(DOCX)

S4 Fig Comparison of Pigment a) Lycopene content b) Chlorophyll a content c) Chlorophyll b content d) Total carotenoids e) Total chlorophyll content.
(DOCX)
