# Supplementary material for: Biochemical profile and bioactive potential of thirteen wild folk medicinal plants from Balochistan, Pakistan
Source: PLoS One. 2020 Aug 18;15(8):e0231612. doi: 10.1371/journal.pone.0231612 (PMC7444594; doi:10.1371/journal.pone.0231612)
Supplement: S1 Fig — Comparison of a) Peroxidase Activity b) Catalase Activity c) Superoxide Dismutase Activity d) Ascorbate Peroxidase Activity e) Alpha-amylase activity f) Esterase activity. (DOCX) [file pone.0231612.s001.docx]

S1 Fig. Comparison of a) Peroxidase Activity b) Catalase Activity c) Superoxide Dismutase Activity d) Ascorbate Peroxidase Activity e) Alpha-amylase activity f) Esterase activity.
