# Supplementary material for: Biochemical profile and bioactive potential of thirteen wild folk medicinal plants from Balochistan, Pakistan
Source: PLoS One. 2020 Aug 18;15(8):e0231612. doi: 10.1371/journal.pone.0231612 (PMC7444594; doi:10.1371/journal.pone.0231612)
Supplement: S2 Fig — Comparison of a) Total Phenolic Contents b) Total Flavonoid c) Ascorbic Acid Content. d) Tannin. (DOCX) [file pone.0231612.s002.docx]

S2 Fig. Comparison of a) Total Phenolic Contents b) Total Flavonoid c) Ascorbic Acid Content d) Tannin.
