# Supplementary material for: Biochemical profile and bioactive potential of thirteen wild folk medicinal plants from Balochistan, Pakistan
Source: PLoS One. 2020 Aug 18;15(8):e0231612. doi: 10.1371/journal.pone.0231612 (PMC7444594; doi:10.1371/journal.pone.0231612)
Supplement: S3 Fig — Comparison of a) Total soluble proteins b) Reducing Sugar c) Total Oxidant Status d) Total Antioxidant Capacity. (DOCX) [file pone.0231612.s003.docx]

S3 Fig. Comparison of a) Total soluble proteins b) Reducing Sugar c) Total Oxidant Status d) Total Antioxidant Capacity.
