# Supplementary material for: Biochemical profile and bioactive potential of thirteen wild folk medicinal plants from Balochistan, Pakistan
Source: PLoS One. 2020 Aug 18;15(8):e0231612. doi: 10.1371/journal.pone.0231612 (PMC7444594; doi:10.1371/journal.pone.0231612)
Supplement: S4 Fig — Comparison of Pigment a) Lycopene content b) Chlorophyll a content c) Chlorophyll b content d) Total carotenoids e) Total chlorophyll content. (DOCX) [file pone.0231612.s004.docx]

S4 Fig. Comparison of Pigment a) Lycopene content b) Chlorophyll a content c) Chlorophyll b content d) Total carotenoids e) Total chlorophyll content.
